# BOOK REVIEW

**DOI:** 10.3402/fnr.v59.30075

**Published:** 2015-11-13

**Authors:** Steve Morrison

**Affiliations:** P2F Ventures Limited, UK

**Clinical aspects of functional foods and nutraceuticals**, edited by Dilip Ghosh, Debasis Bagchi, and Tetsuya Konishi (2015). Boca Raton, FL: CRC Press, Taylor & Francis Group. 452 pp. (Hardback). ISBN 13:978-1-4665-6910-2

In many respects, the functional food space can still be considered an emerging sector. Globally, there is a continued rapid growth in the sales of functional foods and food ingredients but with widely differing regulatory requirements and a multitude of possible routes to market. In Europe, where the new regulatory framework (EU Regulation No.: 1924/2006) has been in place since early 2007, there has only been limited success in gaining approved health claims, and clinical research (human intervention trials) undoubtedly still remains the key driver of success or failure.

This comprehensive book, with contributions from a large number of experienced authors, brings together some of the latest knowledge in the sector in a logical and well-presented format and focuses on the challenges from the perspective of, not only the functional food industry but also its consumers and policy makers. The book is logically organized into six sections, broadly covering the science behind the products, the challenges of innovation in an increasingly regulated domain, the technical challenges inherent in some functional foods, and concludes with a look at future trends.

The introduction to this book covers the development of different dietary guidelines across the globe and also focuses on the ‘barriers’ that future clinical development programs need to overcome in order to succeed. This section concludes with some well-considered, but perhaps rather sobering, thoughts on the costs of delivering quality clinical data versus the prospective rewards that are currently on offer through product commercialization.

Sections II and III look at some individual health benefit areas and the science behind them, demonstrating the clear potential of functional foods in some vital areas of health and citing specific examples of successful past research. The challenges of the probiotic space are perhaps symbolic of the struggles ongoing in the functional food space, and the authors have had a careful look at this sector, acknowledging the widely perceived health benefits associated with these microorganisms and yet recognizing the limited regulatory success achieved so far and the reasons for that apparent contradiction.

Section IV examines the challenges posed by the existing regulatory frameworks, bringing into perspective the prime challenge facing the functional food industry. The evolution of the regulations, as they exist today, is presented in a very clear and concise manner looking at all key global territories, and the primary requirement to present the totality of scientific evidence, which is often challenging in the functional food space, is considered with the authors of the book echoing the calls of the industry itself for greater harmonization of these regulatory guidelines, covering nutrition and heath claims, in the future.

Section VI, the concluding section, looks at the future trends, including a look at the interesting concept of personalized nutrition, which has already gained recognition in the pharmaceutical world and which in the functional food space is as relevant as the interest in modulation of the human microbiome.

This book will provide a valuable resource to all professionals in the functional food space, especially to those who are targeting future health claims and who are seeking to gain an improved understanding of where the functional food sector sits in a dynamic and ever-evolving environment.

*Steve Morrison* P2F Ventures Limited, UK


